# Detection of NO_3_^−^ introduced in plasma-irradiated dry lettuce seeds using liquid chromatography-electrospray ionization quantum mass spectrometry (LC-ESI QMS)

**DOI:** 10.1038/s41598-022-16641-1

**Published:** 2022-07-22

**Authors:** Takamasa Okumura, Pankaj Attri, Kunihiro Kamataki, Naoto Yamashita, Yuichi Tsukada, Naho Itagaki, Masaharu Shiratani, Yushi Ishibashi, Kazuyuki Kuchitsu, Kazunori Koga

**Affiliations:** 1 Faculty of Information Science and Electrical Engineering, Kyushu University, Fukuoka, 819-0395 Japan; 2 Center of Plasma Nano-Interface Engineering, Kyushu University, Fukuoka, 819-0395 Japan; 3 Faculty of Agriculture, Kyushu University, Fukuoka, 819-0395 Japan; 4 Faculty of Science and Technology, Department of Applied Biological Science, Tokyo University of Science, Chiba, 278-8510 Japan; 5 Center for Novel Science Initiatives, National Institutes of Natural Sciences, Tokyo, 105-0001 Japan

**Keywords:** Electrical and electronic engineering, Chemistry

## Abstract

Discharge plasma irradiates seeds with reactive oxygen and nitrogen species (RONS). However, RONS introduced in seeds by plasma irradiation have not been successfully detected thus far. This study provides experimental evidence that nitrate ion NO_3_^−^ is introduced in lettuce seeds as RONS upon irradiation with atmospheric-pressure air dielectric barrier discharge plasma. Plasma irradiation for 5 min promotes seed germination. The components of the plasma-irradiated seeds were examined using electrospray ionization quantum mass spectrometry (ESI QMS), which revealed that the plasma irradiation introduced an ion with a mass of 62 m/z in detectable amounts. This ion was identified as NO_3_^−^ by liquid chromatography (LC), multiple wavelength detector (MWD), and LC-ESI QMS. A one-dimensional simulation at electron temperature T_e_ = 1 eV, electron density N_e_ = 10^13^/m^3^, and gas temperature T_g_ = 300 K indicated the introduction of NO_3_^−^, involving nitric oxide NO. NO_3_^−^ is one of the most important ions that trigger signal transduction for germination when introduced in seeds. The scanning electron microscopy (SEM) images revealed that there was no change on the surface of the seeds after plasma irradiation. Plasma irradiation is an effective method of introducing NO_3_^−^ in seeds in a dry process without causing damage.

## Introduction

The growth improvement effect of plasma irradiation on plants has attracted considerable global attention^1–3^. Thus far, there have been pioneering studies on the improvement of germination and growth^4–19^, control of hormone balance between gibberellic acid (GA) and abscisic acid (ABA) in seeds^20,21^, and improvement of harvest characteristics^22^ using plasma irradiation. Recently, molecular biological studies were conducted to elucidate the mechanisms underlying the biological effects of plasma irradiation^23^. Plasma can irradiate seeds with reactive oxygen and nitrogen species (RONS), photons, ions, and subjects them to electric fields^24^. RONS are involved in a variety of seed processes, including maturation, aging, and germination, followed by seedling growth^25,26^. Exogenous reactive oxygen species (ROS) improves *Zea mays* and *Helianthus annuus* seed germination by inducing GA biosynthesis and ABA catabolism^27^. Colorimetric determination and simulations are useful for estimating the amount of RONS introduced in seeds. However, RONS introduced in seeds by plasma irradiation have not been successfully detected thus far. Considering that many studies deal with the induction of biological responses in seeds upon plasma irradiation, discussions on their underlying mechanisms should be based on the actual particles introduced in the seeds. This study provides experimental evidence that nitrate ions NO_3_^−^ are introduced in seeds as RONS upon plasma irradiation.

We attempted to detect the typical RONS, NO_3_^−^, introduced in seeds upon irradiation with atmospheric-pressure air non-thermal plasma. Seed response to exogenous NO_3_^−^ administration is an important research subject in plant molecular physiology. NO_3_^−^ in plant seeds is responsible for inducing responses such as dormancy break, gene expression regulation, signal transduction, and ABA metabolism resulting from NLP8 binding to the *CYP707A2* promoter^28–32^. Although there are many studies on the changes in genotype and phenotype due to the administration of NO_3_^−^, the molecular mechanism behind the plant’s response to NO_3_^−^ remains unclear^30^. Consequently, this study aimed to experimentally prove that NO_3_^−^ is introduced in seeds by plasma irradiation; a probable mechanism for this introduction is also suggested. It also proposed a new method of administering NO_3_^−^ to seeds in a dry process using plasma.

## Result and discussion

### Germination characteristics

**Figure 1:**
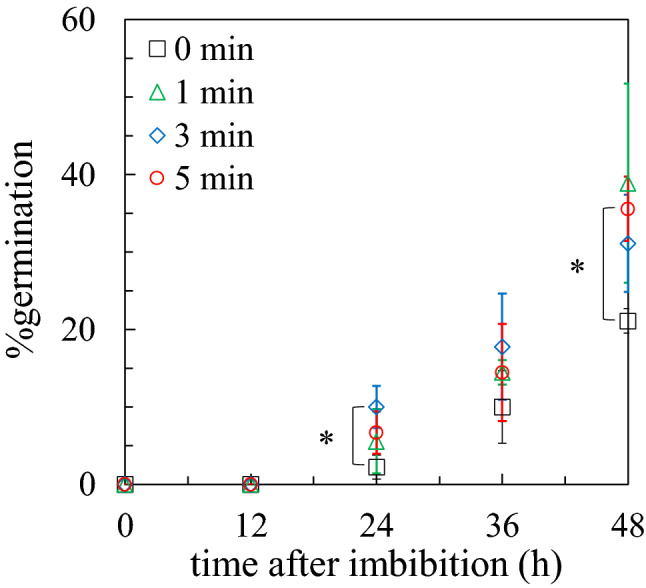
Germination characteristics of lettuce seeds without and with (1, 3, and 5)-min plasma irradiation.

Considering that the introduction of NO_3_^−^ in seeds by plasma irradiation could raise the level of food production, lettuce (*Lactuca sativa* L.), which is the most commonly grown factory-plant^33^, was used as biological material in anticipation of the rapid social implementation of plasma technology. Figure 1 shows the germination characteristics of seeds irradiated with plasma for 1, 3, and 5 min and non-irradiated seeds. This result was obtained using three biological replicates. The marks and error bars indicate average values and standard deviations, respectively. After 24 h, the seeds started to germinate with a relatively large deviation; the %germination was always higher in the seeds with (w/) plasma irradiation than in those without (w/o) plasma irradiation, suggesting that plasma irradiation promotes seed germination. This variability was probably due to the survival strategy of the plant and the amount of RONS introduced by the plasma. As the seed with 5 min of plasma irradiation had a 69% increase in its *p*-value compared to that (0.027) of the seed without plasma irradiation after 48 h upon two-tailed test, a plasma irradiation period of 5 min was decided for further experiments.

### Detection of plasma-induced unknown molecules in seeds

**Figure 2:**
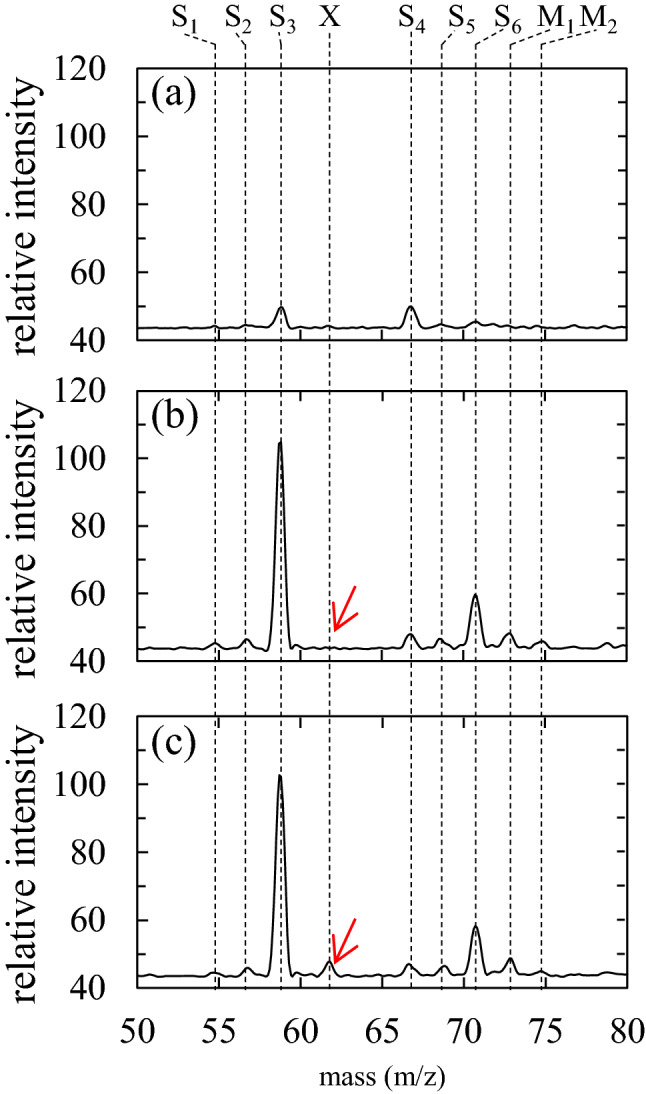
Typical MS spectra of (**a**) blank, and extract of 20 seeds (**b**) without plasma irradiation and (**c**) with 5 min plasma irradiation, obtained by QMS.

Electrospray ionization quantum mass spectrometry (ESI QMS) was used to detect NO_3_^−^ in the seeds. Figure 2 shows the MS spectra of (a) ultra-pure water (blank), (b) the sample without plasma, and (c) the sample with 5 min plasma irradiation. Peaks S_1_, S_2_, S_3_, S_4_, S_5_, and S_6_ are shown in Fig. 2a–c. These peaks are observed even in ultra-pure water (Fig. 2a) and are thus assumed to have originated from the LC-QMS system. The intensities of peaks S_1_ to S_6_ in Fig. 1b and c are larger than those in Fig. 2a. This might be due to the matrix effect of seed contaminants. Conversely, the M_1_ and M_2_ peaks are found only for the seed extract in Fig. 1b and c, indicating that these two peaks originate from the seed material. The intensities of the M_1_ and M_2_ peaks in Fig. 2b are almost the same as those in Fig. 2c, indicating that the concentrations of the sample w/o and w/ plasma are the same and that plasma irradiation does not change the substantial composition in the mass range of 50–80 m/z. Nevertheless, a peak X at 62 m/z (indicated with an arrow) is clearly observed in Fig. 2c.

**Figure 3:**
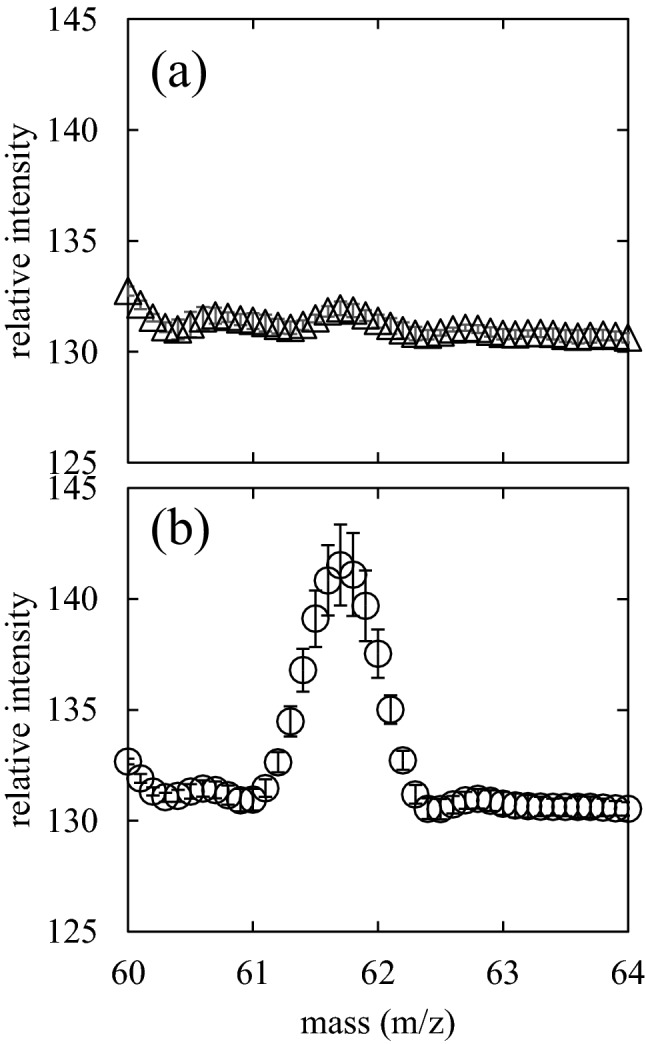
MS spectrum of 60–64 m/z of extract of 20 seeds (**a**) without plasma irradiation and (**b**) with 5 min plasma irradiation, obtained by QMS mode. Three shots were integrated. Marks and error bars show mean values and standard deviations of triplicate, respectively.

Further experiments were conducted to evaluate the reproducibility with five biological replicates. The MS spectra in the 60–64 m/z mass region of 20 seeds w/o plasma irradiation are shown in Fig. 3a, and those w/plasma irradiation are shown in Fig. 3b. We performed three measurements per replicate and obtained the integrated values. The marks and error bars indicate average values and standard deviations, respectively. The relative intensity at 62 m/z without plasma irradiation was 131.96 ± 0.31, which was almost the same as the baseline (131) but was 141.53 ± 1.83 for seeds with plasma irradiation. The peak area was significantly higher in the extract w/ plasma (*p* = 0.00012 as determined by a two-tailed *t*-test) than in the extract w/o plasma irradiation. These results strongly indicate that 62 m/z molecules generated by plasma irradiation were introduced in the seeds. Considering that the optical emission spectra of scalable dielectric barrier discharge (SDBD) plasma show NO (200–250 nm)^34^, a major candidate for peak X is nitrate ion (NO_3_^−^) among the long-lifetime species in air plasmas. NO_3_^−^ can be generated from the electron impact ionization reactions of N_2_, O_2_, and H_2_O in the gas phase as shown in the reactions (R1)–(R7)^35^

R1 $${\text{e }} + {\text{ N}}_{{2}} \to {\text{e }} + {\text{ 2N}}$$

R2 $${\text{e }} + {\text{ H}}_{{2}} {\text{O}} \to {\text{e }} + {\text{ H }} + {\text{ OH}}$$

R3 $${\text{e }} + {\text{ O}}_{{2}} \to {\text{ 2O }} + {\text{ e}}$$

R4 $${\text{N }} + {\text{ OH}} \to {\text{NO }} + {\text{ H}}$$

R5 $${\text{NO }} + {\text{ O}} \to {\text{NO}}_{{2}}$$

R6 $${\text{NO}}_{{2}} + {\text{ e}} \to {\text{NO}}_{{2}}^{ - }$$

R7 $${\text{NO}}_{{2}} + {\text{ NO}}_{{2}}^{ - } \to {\text{NO}}_{{3}}^{ - } + {\text{ NO}}$$

### Identification of ion 62 m/z

**Figure 4:**
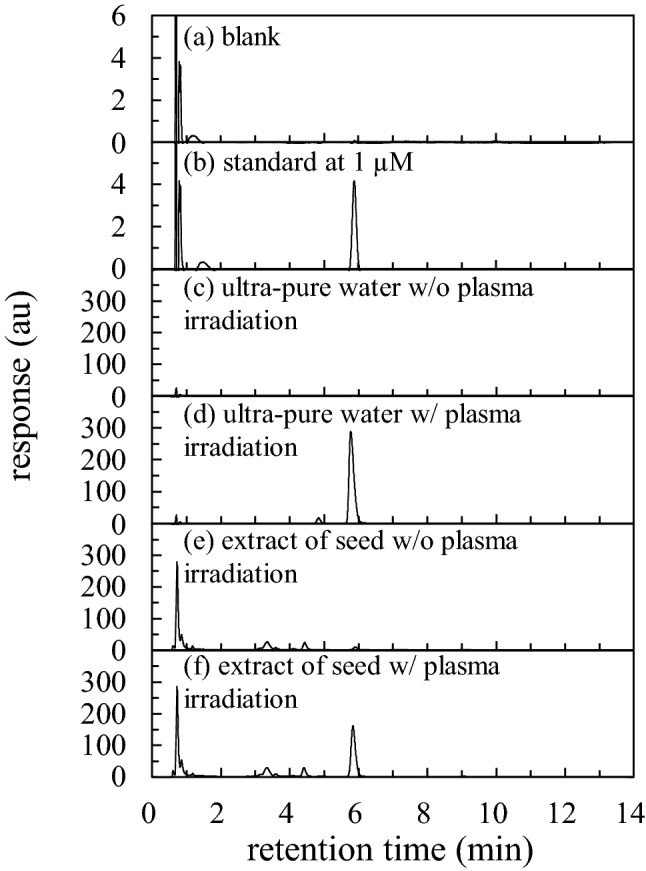
MWD chromatograms of 210 nm of (**a**) blank, (**b**) standard solution of NO_3_^−^ at 1 µM of concentration, ultra-pure water (**c**) without plasma irradiation and (**d**) with plasma irradiation for 300 s, and extract of seed (**e**) without plasma irradiation and (**f**) with plasma irradiation for 5 min.

A liquid chromatography (LC)-multiple wavelength detector (MWD) and LC-ESI QMS were used to identify the 62 m/z peak. Chromatography is effective in measuring the amount of NO_3_^−^ in seed extract from impurities such as enzymes, proteins, and organic metals therein. The nitrate ion absorbs a wavelength of ~ 224 nm, and its isosbestic point is 215 nm^36^. A wavelength of 210 nm was used for MWD to avoid interference by peptide bonds of protein, which absorb at 228 nm, in seed extract^37^. It has been reported that NO_3_^−^ is a major ion found in plasma-irradiated water^1,38^; hence, ultra-pure water was also irradiated with plasma and used for the analysis. Figure 4 shows the MWD chromatogram at 210 nm for (a) blank (ultra-pure water), (b) standard solution of NO_3_^−^ at 1 µM, (c) ultra-pure water without plasma irradiation, (d) ultra-pure water with plasma irradiation, (e) seed extract without plasma irradiation, and (f) seed extract with plasma irradiation. The disturbance observed within 2 min is due to the injection shock and is thus neglected. Figure 4a shows no peaks after 2 min in the blank (ultra-pure water used for preparing the seed extraction). Ultra-pure water was used to dilute the standard reagent NO_3_^−^. A peak corresponding to NO_3_^−^ appears at 5.84 min in the MWD, as shown in Fig. 4b. Although there is no NO_3_^−^ peak in Fig. 4c, we can find a peak with high intensity in Fig. 4d and f, showing that NO_3_^−^ is introduced in ultra-pure water and seeds by plasma irradiation. A small NO_3_^−^ peak was also observed in seeds without plasma irradiation. The seed extract had several peaks at 3–5 min, as shown in Fig. 4e and f, and the areas of these peaks are almost the same, whereas the NO_3_^−^ peak areas are significantly different from each other, i.e., 81.05 and 1486.04 for w/o and w/plasma, respectively. According to the Lambert–Beer law, the amount of NO_3_^−^ seems to be considerably high in the extracts of seeds subjected to plasma irradiation. However, it cannot be denied that plasma irradiation may have produced an ion in the seed, which may have absorbed UV at approximately 210 nm with the same retention time as that of NO_3_^−^. Therefore, we performed an LC-ESI QMS experiment, corresponding to the results of Figs. 2, 3, and 4.

**Figure 5:**
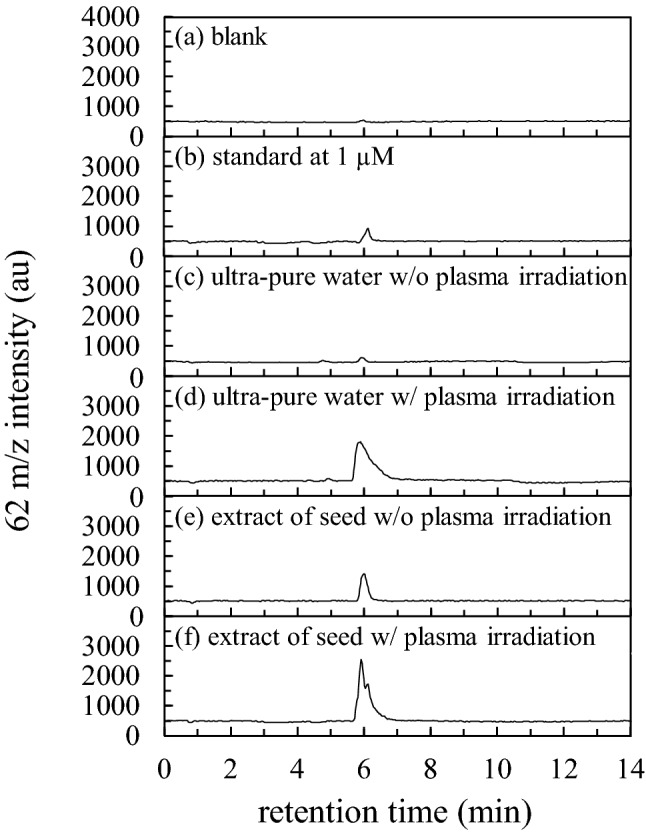
EIC of 62 m/z of (**a**) blank, (**b**) standard solution of NO_3_^−^ at 1 µM of concentration, ultra-pure water (**c**) without plasma irradiation and (**d**) with plasma irradiation for 5 min, and extract of seed (**e**) without plasma irradiation and (**f**) with plasma irradiation for 5 min.

The isotope distribution of a molecule to be analyzed is unique to its structure and very effective in identifying those that indicate the detected mass. As stable isotopes are present in each of the nitrogen and oxygen atoms, the proportion of NO_3_^−^ was calculated using the isotope distribution calculator (Agilent MassHunter Workstation Data Analysis Core; Version 10.0); using this we can estimate the mass that is dominantly detected for NO_3_^−^. The calculated values were 61.99 m/z, 62.99 m/z, 63.99 m/z, and 64.99 m/z of mass corresponding to 98.91%, 0.47%, 0.61%, and 0.00% of abundance, respectively, indicating that approximately 99% of NO_3_^−^ existing in nature gives a mass of 62 m/z. Consequently, to identify 62 m/z of NO_3_^−^, a chromatogram of 62 m/z was obtained for the standard reagent of NO_3_^−^ and extract of plasma-irradiated seeds. The mass resolution of the QMS was within 1 m/z. The coincidence of these retention times strongly indicated that they were the same substances. Although the signal intensity saturates at high concentrations for the extracted ion chromatogram (EIC), it can detect substances in smaller amounts compared to that by MWD. Figure 5 shows the ESI of 62 m/z for (a) blank, (b) standard solution of NO_3_^−^ at 1 µM, (c) ultrapure water without plasma irradiation, (d) ultrapure water with plasma irradiation, (e) seed extract without plasma irradiation, and (f) seed extract with plasma irradiation. Figure 5 was obtained from the same sample shown in Fig. 4 to eliminate the contamination and concentration variation within the dilution processes. There is no peak in the blank, as shown in Figs. 5a and 4a. The standard reagent showed a peak at 5.8–6.3 min retention time, as shown in Fig. 5b. Figure 5c and d show ultra-pure water with and without plasma, respectively. During the plasma irradiation, ultrapure water was placed in a quartz Petri dish. A peak does not appear in the ultra-pure water simply placed in the quartz Petri dish (without irradiation), as shown in Fig. 5c, whereas a large peak appears at 5.8–7.0 min retention time when the same sample is irradiated with plasma, as shown in Fig. 5d. This retention time was in the same range as that of the standard reagent. Thus, the 62 m/z ion obtained from plasma irradiation was identified as NO_3_^−^. For the seed extract, peaks were found in the same retention time region as that shown in Fig. 5e and f, suggesting that NO_3_^−^ was already present in the seed prior to plasma irradiation; its amount increased upon plasma irradiation. The standard deviations (±) in the mean peak areas for the seed extract were 4.6 × 10^3^ ± 660.5 and 3.9 × 10^4^ ± 4154.9 with and without plasma irradiation, respectively. The statistical *t*-test showed that *p* = 0.0006. Although it is clear that NO_3_^−^ was introduced in the seeds by plasma irradiation, it is also interesting that NO_3_^−^ was already present in the seeds at detectable levels prior to plasma irradiation. This method may be useful for measuring the amount of NO_3_^−^ originally present in untreated seeds.

### Introduction pathway of NO_3_^−^ into seeds by plasma irradiation

The feasibility of NO_3_^−^ introduction in seeds by air SDBD plasma irradiation at atmospheric pressure was assessed by 1D simulation using COMSOL with 624 generation and decomposition reaction equations, summarized by Sakiyama et al.^39^, wherein the electron temperature Te = 1 eV, electron density Ne = 10^13^/m^3^, gas temperature Tg = 300 K, and plasma width Lp = 0.1 mm for a time duration of 1000 s. The simulation results present the density of NO_3_^−^ and that of the reactive species involved in its generation at a point 5 mm away from the plasma region corresponding to the seed position. Table 1 shows the simulation results of the density at a certain point, e.g., seeds placed under the SDBD electrode after 1000 s, for NO_3_^−^ and the reactive species that participate in its generation through the following reactions^39–42^:

R8 $${\text{NO}}_{{2}}^{ - } + {\text{ HNO}}_{{3}} \to {\text{ NO}}_{{3}}^{ - } + {\text{ HNO}}_{{2}} \;\;\;\;\;{1}.{6} \times {1}0^{{ - {15}}}$$

R9 $${\text{NO}}_{{2}}^{ - } + {\text{ N}}_{{2}} {\text{O}}_{{5}} \to {\text{NO}}_{{3}}^{ - } + {\text{ NO}}_{{3}} + {\text{ NO}} \;\;\;\;\;\; {7} \times {1}0^{{ - {16}}}$$

R10 $${\text{O}}_{{2}}^{ - } + {\text{ NO}}_{{3}} \to {\text{NO}}_{{3}}^{ - } + {\text{ O}}_{{2}} \;\;\;\;\;\;{5} \times {1}0^{{ - {16}}}$$

R11 $${\text{O}}_{{3}}^{ - } + {\text{ NO}}_{{3}} \to {\text{NO}}_{{3}}^{ - } + {\text{ O}}_{{3}} \;\;\;\;\;{5} \times {1}0^{{ - {16}}}$$

R12 $${\text{NO}}_{{2}}^{ - } + {\text{ NO}}_{{3}} \to {\text{NO}}_{{3}}^{ - } + {\text{ NO}}_{{2}} \;\;\;\; {5} \times {1}0^{{ - {16}}}$$

R13 $${\text{NO}}^{ - } + {\text{ NO}}_{{3}} \to {\text{NO}}_{{3}}^{ - } + {\text{ NO}} \;\;\;\;\;\; {3} \times {1}0^{{ - {16}}}$$

R14 $${\text{O}}_{{2}}^{ - } + {\text{ HNO}}_{{3}} \to {\text{NO}}_{{3}}^{ - } + {\text{ HO}}_{{2}} \;\;\;\;\;\; {2}.{8} \times {1}0^{{ - {16}}}$$

R15 $${\text{O}}_{{3}}^{ - } + {\text{ NO}}_{{2}} \to {\text{NO}}_{{3}}^{ - } + {\text{ O}}_{{2}} \;\;\;\;\;\;\;\;\;{2} \times {1}0^{{ - {17}}}$$

R16 $${\text{NO}}_{{2}}^{ - } + {\text{ O}}_{{3}} \to {\text{NO}}_{{3}}^{ - } + {\text{ O}}_{{2}} \;\;\;\;\;{1}.{8} \times {1}0^{{ - {17}}}$$

R17 $${\text{O}}_{{3}}^{ - } + {\text{ NO}} \to {\text{NO}}_{{3}}^{ - } + {\text{O}}\;\;\;\;\;\;\;\;{1} \times {1}0^{{ - {17}}}$$

R18 $${\text{NO}}_{{2}}^{ - } + {\text{ NO}}_{{2}} \to {\text{NO}}_{{3}}^{ - } + {\text{ NO}} \;\;\;\;\;\; {4} \times {1}0^{{ - {18}}}$$

R19 $${\text{NO}}_{{2}}^{ - } + {\text{ N}}_{{2}} {\text{O}} \to {\text{NO}}_{{3}}^{ - } + {\text{ N}}_{{2}} \;\;\;\;\;\; {5} \times {1}0^{{ - {19}}}$$

**Table 1:** 1D simulation result for density of NO_x_^−^ relating reactive species at a certain point such as seeds after 1000 s.

Chemical formula	Density (/m^3^)
NO_3_^−^	3.9 × 10^3^
NO	6.6 × 10^18^
NO_2_	1.4 × 10^10^
NO_2_^−^	1.1 × 10^2^
NO_3_	1.0 × 10^2^
HNO_3_	1.2 × 10^–1^
HNO_2_	2.4 × 10^–10^
N_2_O_5_	2.2 × 10^–29^

The unit of the rate constant corresponding to the aforementioned reactions is (m^3^/s). Table 1 shows that NO_3_^−^ is directly transported to seeds along with the simultaneous transportation of the reactive species participating in its generation, such as NO, NO_2_, NO_2_^−^, NO_3_, HNO_3_, HNO_2_, and N_2_O_5_. The DBD plasma is accompanied by a streamer discharge. The reduced electric field E/N in the initial DBD plasma is approximately 100 Td and that in the typical streamer channel is < 30 Td^43^. In this state, the electron temperature T_e_ < 2.7 eV, and such electrons are mainly consumed for the vibration excitation of N_2_ and O_2_ in the air at atmospheric pressure. The vibrational states of N_2_ and O_2_ contribute to the formation of NO through the following reactions, commonly known as the Zeldovich mechanism^44^. The activation energy *E*_a_ and enthalpy of reaction Δ*H* both are nearly 3 eV/mol for R20, and 0.3 eV/mol and − 1 eV/mol for R21, respectively.

R20 $${\text{O }}\left( {^{{3}} P} \right) \, + {\text{ N}}_{{2}} *(^{{1}} \Sigma_{{\text{g}}}^{ + } ,v) \, \to {\text{ NO}}(^{{2}} \Pi ) \, + {\text{ N}}\left( {^{{4}} {\text{S}}} \right),$$

R21 $${\text{N }} + {\text{ O}}_{{2}} \to {\text{ NO }} + {\text{ O}}$$

Hence, NO shows the highest density (6.6 × 10^18^/m^3^) in Table 1. High-density NO may react with the H_2_O in the seeds. According to Lukes et al., NO leads to NO_3_^−^ through the following reaction^45^.

R22 $${\text{2NO }} + {\text{ O}}_{{2}} + {\text{ H}}_{{2}} {\text{O }} \to {\text{ 2H}}^{ + } + {\text{ NO}}_{{2}}^{ - } + {\text{ NO}}_{{3}}^{ - }$$

The lettuce seeds used in this study had an average weight ± standard deviation of approximately 0.8 ± 0.04 mg/seed (n = 8). Water content in more than 1250 seeds was measured and found to be 4.705 ± 0.503 wt% (n = 4). Using these mean values, the number of water molecules in the seed, ${n}_{\mathrm{H}2\mathrm{O}}$, was derived by Eq. (1):

1 $${n}_{\mathrm{H}2\mathrm{O}}=\frac{w{N}_{\mathrm{A}}}{M}$$

where $w$ is the weight of H_2_O in the seed (37.64 µg/seed), ${N}_{\mathrm{A}}$ is the Avogadro constant (6.022 × 1023/mol), and $M$ is the H_2_O molar mass (18 g/mol). The seed area was 0.393 ± 0.00374 × 10^–6^ m^2^ (n = 290), and the seed volume was derived to be 0.393 × 10^–9^ m^3^/seed, assuming a thickness of 1 mm. The volume of NO $({n}_{\mathrm{NO}})$ and H_2_O (${n}_{\mathrm{H}2\mathrm{O}})$ in the seed were 2.594 × 10^9^ and 1.259 × 10^18^, respectively. When NO reacted with H_2_O in the seed, two molecules of NO and one molecule of H_2_O were consumed to bear NO_2_^−^ and NO_3_^−^, as shown in R22. Therefore, even if NO reacted completely with H_2_O, sufficient H_2_O would remain in the seeds. Considering the high density of NO in a stable (O_2_ and H_2_O)-rich environment, it is concluded that the introduction pathway of NO_3_^−^ into seeds by plasma irradiation mainly involved reaction (R22). On the other hand, according to Sakiyama et al*.*^39^, NO_2_^−^ reacts with NO and produces NO^−^ and NO_2_ by the following reaction^46^.

R23 $${\text{NO}}_{{2}}^{ - } + {\text{ NO }} \to {\text{ NO}}^{ - } + {\text{ NO}}_{{2}}$$

NO^−^ reacts with M, O_2_, and NO and produces NO, O_2_^−^, and e by the following reactions^47^.

R24 $${\text{NO}}^{ - } + {\text{ M }} \to {\text{ NO }} + {\text{ M }} + {\text{ e}}$$

R25 $${\text{NO}}^{ - } + {\text{ O}}_{{2}} \to {\text{ NO }} + {\text{ O}}_{{2}}^{ - }$$

R26 $${\text{NO}}^{ - } + {\text{ NO }} \to {\text{ NO }} + {\text{ NO }} + {\text{ e}}$$

NO is involved in R22. O_2_^−^ reacts with N_2_ and O_2_ and produces e by the following reactions^41^.

R27 $${\text{O}}_{{2}}^{ - } + {\text{ N}}_{{2}} \to {\text{ N}}_{{2}} + {\text{ O}}_{{2}} + {\text{ e}}$$

R28 $${\text{O}}_{{2}}^{ - } + {\text{ O}}_{{2}} \to {\text{ O}}_{{2}} + {\text{ O}}_{{2}} + {\text{ e}}$$

The electron e reacts with NO and O_2_ and produces NO^−^ and O_2_^−^ by the following reactions^39,41^.

R29 $${\text{e }} + {\text{ NO }} + {\text{ M }} \to {\text{ NO}}^{ - } + {\text{ M}}$$

R30 $${\text{e }} + {\text{ O}}_{{2}} \to {\text{ O}}_{{2}}^{ - }$$

NO^−^ and O_2_^−^ are involved by reactions of R24-26 and R27, 28, respectively.

### Assessment of seed surface before and after plasma irradiation

**Figure 6:**
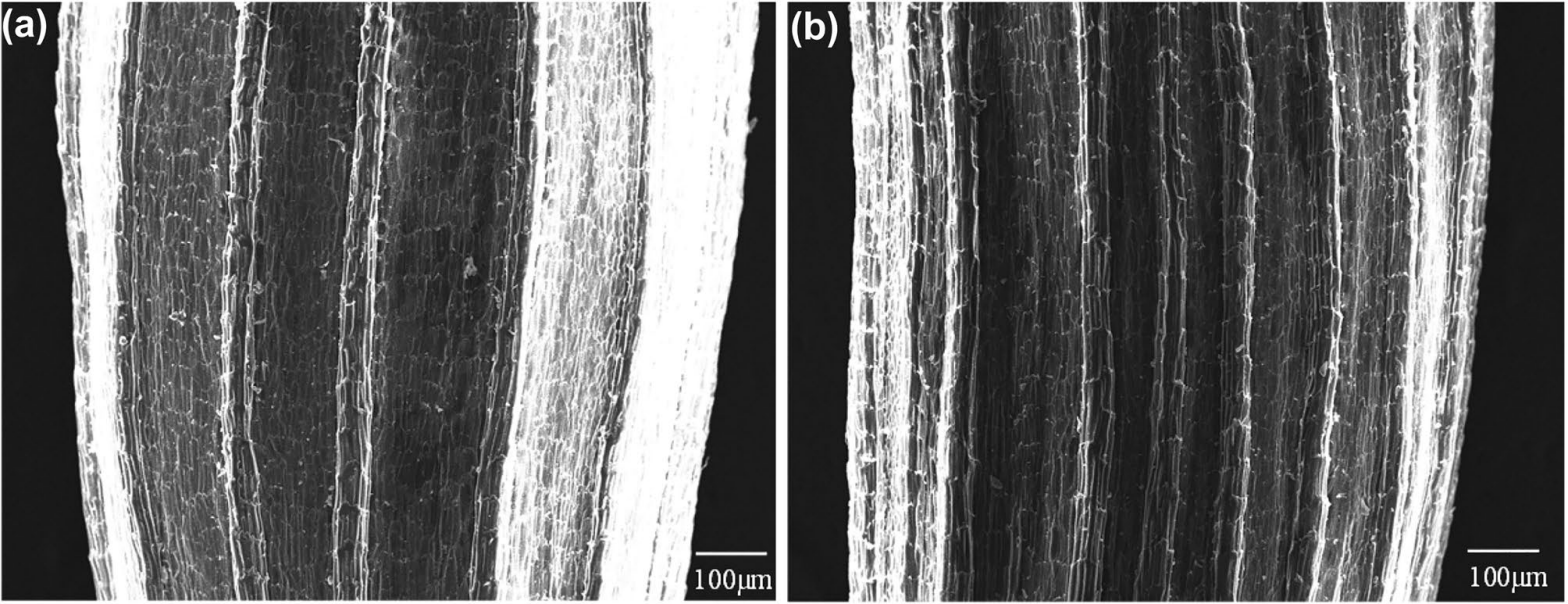
SEM image of the same seed (**a**) before plasma irradiation and (**b**) after plasma irradiation for 10 min.

Figures 2 and 4 suggest that plasma irradiation did not affect the qualitative composition of the seeds. Therefore, the physical effects of plasma irradiation on the surface conditions of the seed coat were investigated using the scanning electron microscopy (SEM) images. Figure 6 shows a typical SEM image obtained (a) before and (b) after the plasma irradiation. The observed surface was the side on which the plasma was irradiated. The seeds were irradiated with plasma for 10 min, which was twice as long as the conditioning period of the seeds for mass spectroscopy. Lettuce seeds are composed of an outer layer, called the pericarp as the seed coat and integument, endosperm, and embryo as the internal organelle^48^. A typical pericarp was observed in the seeds used in this study, as shown in Fig. 6a. The pericarp remained intact after the plasma irradiation as shown in Fig. 6b. It is composed of a complex, dynamic extracellular matrix consisting of a network of carbohydrate polymers (cellulose, hemicellulose, and pectin) and structural proteins^48^. The chemical species produced by the plasma, such as O_3_, may cleave or dimerize the carbon–carbon double bond (C=C) of alkene^49^, including the unsaturated fatty acids in the lettuce seeds, followed by a C=C loss. Raman spectroscopy was used to assess such a chemical damage on the seed by plasma irradiation. A typical Raman spectrum at 980–1970 cm^−1^ of lettuce seeds w/o plasma is shown in Fig. S1. We can find a peak at 1609 cm^−1^, corresponding to C=C peak that is assigned to lignin and flavonoid (Fig. S1).

Raman spectroscopy revealed that the carbon–carbon double bond (C=C) peak assigned to lignin and flavonoid did not change after plasma irradiation for 5 and 10 min (Fig. S2). The chemical species produced by the plasma, such as O_3_, may cleave or dimerize C=C of alkene^49^, including the unsaturated fatty acids in the lettuce seeds, followed by a C=C loss, but such an effect is undetectable. These results suggest that plasma irradiation can introduce NO_3_^−^ without chemical or physical damage to the seeds.

## Conclusion

In this study, we developed a method for detecting NO_3_^−^ introduced in dry lettuce seeds by plasma irradiation using LC-ESI QMS. To date, only the amount of RONS in the gas phase and solution has been evaluated; this is the first time that plasma-introduced RONS in the treated seeds has been detected. As the molecular mechanism of the seed’s cell response to NO_3_^−^ exogenously introduced in it is still under investigation, NO_3_^−^ remains one of the most important molecules in terms of seed physiology^30^. Thus far, the only method of exogenous administration of NO_3_^−^ in seeds has been to immerse them in a KNO_3_ solution. However, plasma allows us to introduce NO_3_^−^ without the requirement of water or counterions. The quantitative evaluation of the amount of NO_3_^−^ introduced in seeds by plasma irradiation is decisive in the contribution of plasma science to plant molecular biology and the quantitative discussion of the germination induction mechanism by plasma irradiation.

## Methods

### Seed treatment and germination test

Lettuce seed (*Lactuca sativa* L.) was purchased from Asahi Farm, Japan. The experimental use of plant materials was performed with the permission of Asahi Farm. The seeds were irradiated with plasma using a scalable dielectric discharge (SDBD) electrode^21^. The characteristics of the plasma used in this study have been discussed in a previous paper^9^. The plasma irradiation was performed at 24 °C using 55% Rh. For NO_3_^−^ detection, 80 lettuce seeds were irradiated simultaneously under a single plasma exposure. The irradiation time was determined to be 5 min based on the germination characteristics of seeds upon 0, 1, 3, and 5 min of plasma irradiation. The germination test was performed with 30 seeds sown on a paper filter in a petri dish, with 3 mL of tap water in a climate chamber at 22 °C under a light and dark cycle of 12 h each day. The number of germinated seeds was counted every 12 h after their imbibition for 48 h. The %germination was calculated by dividing the number of germinated seeds by the total number of seeds in the Petri dish. All methods were performed in accordance with the relevant guidelines and regulations.

### Sample preparation for mass spectrometry

Twenty seeds were used to obtain a seed extract sample in the following procedure: The seeds were pulverized with beads in a 2.0 mL-tube (Eppendorf) using an automatic grinder at 2800 rpm for 3 min. Seed extraction was performed by shaking the pulverized sample with 50 µL of ultrapure water per mg of the sample at room temperature for 120 min under dark conditions. The seed extract was filtered using a membrane filter to remove the particles.

### Mass spectrometry

We used ESI QMS (G6470A, Agilent) to detect NO_3_^−^ in the seeds, with a mobile phase of 3.75 mM ammonium acetate in 70% H_2_O/30% acetonitrile. The mobile phase profile was 0.2 mL/min for ESI QMS and 0.4 mL/min for LC-ESI QMS. The injection volume was 5 µL. Ionization was performed in the negative mode of ESI with a 4000 V capillary voltage, 300 °C turbo gas temperature; and 50 V fragmentor voltage. Agilent Infinity II including LC (G7116A, Agilent) – MWD (G7165A, Agilent) were used for the identification and the quantitative analysis. Acclaim Trinity (P1, Thermo Fisher) was used as a column for LC. LC grade ultrapure water was used for the sample dilution. Standard reagent NO_3_^−^ was purchased from FUJIFILM Wako.

### 1D simulation

The 1D numerical model was developed using COMSOL Multiphysics 5.4, a multipurpose simulation software. Electrons exist in the plasma region at a constant number density of 10^15^ m^−3^, as described in our earlier work^35^. We considered the particle distribution only in the x-direction (5 mm), normal to the surface of the seeds. We assumed T_e_ = 1 eV and T_g_ = 300 K. For the simulation, H_2_O, O_2_, and N_2_ were in fractions of 0.01, 0.21, and 0.78, respectively. As mentioned in an earlier article, a coefficient value of 10^−5^ m^2^/s was used for this species^39^. The simulation was performed for 1000 s to determine the diffusion of the reactive species. All reaction pathways and rate constants were taken from the work of Sakiyama et al.^39^.

### SEM

Scanning electron microscopy (SEM) images were obtained using Hitachi S-3400 N, which has an emission current of 93 μA and acceleration voltage of 15 kV.

## Supplementary Information

Supplementary Information.

## Data Availability

The datasets used and/or analysed during the current study available from the corresponding author on reasonable request.
