# Supplementary material for: Detection of NO3− introduced in plasma-irradiated dry lettuce seeds using liquid chromatography-electrospray ionization quantum mass spectrometry (LC-ESI QMS)
Source: Sci Rep. 2022 Jul 22;12:12525. doi: 10.1038/s41598-022-16641-1 (PMC9307838; doi:10.1038/s41598-022-16641-1)
Supplement: Supplementary file 1 — Supplementary Information. [file 41598_2022_16641_MOESM1_ESM.pdf]

Supporting information

Detection of NO_3^- introduced in plasma-irradiated dry lettuce seeds using liquid chromatography-electrospray ionization quantum mass spectrometry (LC-ESI QMS)

*Takamasa Okumura[1], Pankaj Attri[2], Kunihiro Kamataki[3], Naoto Yamashita[4], Yuichi Tsukada[5], Naho Itagaki[6], Masaharu Shiratani[7], Yushi Ishibashi[8], Kazuyuki Kuchitsu[9] and Kazunori Koga[10]

*Corresponding author

1 Faculty of Information Science and Electrical Engineering, Kyushu University, Fukuoka 819-0395, Japan; t.okumura@plasma.ed.kyushu-u.ac.jp

2 Faculty of Agriculture, Kyushu University, Fukuoka 819-0315; attri.pankaj.486@m.kyushu-u.ac.jp

3 Faculty of Information Science and Electrical Engineering, Kyushu University, Fukuoka 819-0395, Japan; kamataki@plasma.ed.kyushu-u.ac.jp

4 Faculty of Agriculture, Kyushu University, Fukuoka 819-0315; n.yamashita@plasma.ed.kyushu-u.ac.jp

5 Center of Plasma Nano-interface Engineering, Kyushu University, Fukuoka 819-0395, Japan; ytsukada@ifrc.kyushu-u.ac.jp

6 Faculty of Agriculture, Kyushu University, Fukuoka 819-0315, Japan; itagaki@ed.kyushu-u.ac.jp

7 Faculty of Agriculture, Kyushu University, Fukuoka 819-0315, Japan; siratani@ed.kyushu-u.ac.jp

8 Faculty of Agriculture, Kyushu University, Fukuoka 819-0315, Japan; yushi@agr.kyushu-u.ac.jp

9 Faculty of Science and Technology, Department of Applied Biological Science, Tokyo University of Science, Chiba 278-8510, Japan; kuchitsu@rs.tus.ac.jp

10 Center for Novel Science Initiatives, National Institute of Natural Science, Tokyo 105-0001, Japan; Faculty of Information Science and Electrical Engineering, Kyushu University, Fukuoka 819-0395, Japan; koga@ed.kyushu-u.ac.jp

Table and Figure Captions for supporting files

Table S1: Raw data of germination characteristics of lettuce seeds with (0, 1, 3, and 5)-min plasma irradiation.

Table S2: Raw data of MS spectrum of 60–64 m/z of extract of 20 seeds without plasma irradiation, obtained by QMS mode. Three shots were integrated. Marks and error bars show mean values and standard deviations of triplicate, respectively.

Table S3: Raw data of MS spectrum of 60–64 m/z of extract of 20 seeds with 5 min plasma irradiation, obtained by QMS mode.

Figure S1: Typical Raman spectrum at 980–1970 cm^{-1} of lettuce seeds.

Figure S2: Raman peak intensity at 1609 cm^{-1}, corresponding to carbon–carbon double bond (C=C) peak assigned to lignin and flavonoid[1].

Table S1

Time of plasma irradiation (min)	Replication	0h	12h	24h	36h	48h
0	1	0.0	0.0	0.0	6.7	23.3
	2	0.0	0.0	3.3	16.7	20.0
	3	0.0	0.0	3.3	6.7	20.0
1	1	0.0	0.0	0.0	13.3	26.7
	2	0.0	0.0	6.7	16.7	56.7
	3	0.0	0.0	10.0	13.3	33.3
3	1	0.0	0.0	6.7	10.0	26.7
	2	0.0	0.0	13.3	26.7	40.0
	3	0.0	0.0	10.0	16.7	26.7
5	1	0.0	0.0	10.0	23.3	40.0
	2	0.0	0.0	3.3	10.0	30.0
	3	0.0	0.0	6.7	10.0	36.7

Table S2

Data point	Mass (m/z)	w/o plasma_1			Integration	w/o plasma_2			Integration	w/o plasma_3			Integration	w/o plasma_4			Integration	w/o plasma_5			Integration
		1st	2nd	3rd		1st	2nd	3rd		1st	2nd	3rd		1st	2nd	3rd		1st	2nd	3rd	
0	60	44.56812	43.74298	44.20959	132.5207	44.20969	44.71808	44.13621	133.064	43.98931	44.33523	44.36483	132.6894	44.63219	44.05456	43.876	132.5628	44.61454	44.04432	44.23523	132.894
1	60.1	44.4266	43.93357	43.99096	132.3511	43.99293	44.39528	43.88779	132.276	43.69854	44.13497	44.01819	131.8517	44.43892	43.82893	43.70399	131.9718	44.23028	43.74959	44.23619	132.2161
2	60.2	44.07587	43.90957	43.76387	131.7493	43.87233	44.07001	43.69346	131.6358	43.5544	43.95015	43.73398	131.2385	44.17861	43.73904	43.5219	131.4395	43.94167	43.55607	44.14112	131.6389
3	60.3	43.62297	43.72356	43.57681	130.9233	43.93067	43.93033	43.62256	131.4836	43.52757	43.77672	43.58553	130.8898	43.89208	43.75078	43.41527	131.0581	43.75372	43.48879	43.96621	131.2087
4	60.4	43.30791	43.57094	43.47281	130.3517	44.05831	44.02091	43.67675	131.756	43.57711	43.68207	43.61759	130.8768	43.71853	43.84354	43.47105	131.0331	43.68195	43.58093	43.79591	131.0588
5	60.5	43.28405	43.53339	43.48909	130.3065	44.09622	44.21807	43.7874	132.1017	43.64938	43.70938	43.80058	131.1593	43.73447	43.97373	43.66141	131.3696	43.72092	43.77526	43.72385	131.22
6	60.6	43.43792	43.54829	43.64404	130.6302	44.0054	44.32981	43.89785	132.2331	43.69896	43.82906	43.97525	131.5033	43.87383	44.02961	43.83443	131.7379	43.79672	43.93671	43.75783	131.4913
7	60.7	43.51529	43.58255	43.90718	131.005	43.85987	44.26829	43.98657	132.1147	43.70667	43.96244	43.9597	131.6288	44.00476	43.93615	43.85553	131.7964	43.83424	43.97836	43.82532	131.6379
8	60.8	43.4021	43.68601	44.17041	131.2585	43.73812	44.15942	44.02293	131.9205	43.6767	44.03344	43.75819	131.4683	44.02605	43.77599	43.73757	131.5396	43.85869	43.93977	43.86835	131.6668
9	60.9	43.22471	43.84556	44.29585	131.3661	43.66944	44.17487	43.95864	131.803	43.61459	43.99908	43.55449	131.1682	43.9243	43.70631	43.61765	131.2483	43.94697	43.90208	43.88411	131.7332
10	61	43.16695	43.9445	44.21257	131.324	43.65494	44.30893	43.79599	131.7599	43.52593	43.88351	43.50511	130.9146	43.78067	43.75627	43.58192	131.1189	44.07452	43.88073	43.87379	131.829
11	61.1	43.27296	43.88697	43.99597	131.1559	43.68039	44.38839	43.63146	131.7002	43.42786	43.76247	43.60123	130.7916	43.6923	43.79838	43.576	131.0667	44.11278	43.83587	43.82788	131.7765
12	61.2	43.44618	43.72413	43.80839	130.9787	43.71835	44.29688	43.5821	131.5973	43.34399	43.70531	43.7361	130.7854	43.67895	43.73512	43.52755	130.9416	44.00568	43.74133	43.77423	131.5212
13	61.3	43.55834	43.60153	43.7646	130.9245	43.7504	44.10117	43.68991	131.5415	43.30373	43.74092	43.82684	130.8715	43.69735	43.65004	43.48788	130.8353	43.8659	43.63837	43.78023	131.2845
14	61.4	43.56594	43.62494	43.8592	131.0501	43.77597	43.96129	43.88436	131.6216	43.34103	43.84948	43.87337	131.0639	43.75401	43.70668	43.56395	131.0246	43.82693	43.6002	43.85661	131.2837
15	61.5	43.52384	43.75703	44.01135	131.2922	43.81725	43.95951	44.02214	131.7989	43.4693	43.99266	43.93098	131.3929	43.90419	43.9535	43.7305	131.5882	43.89005	43.65762	43.93382	131.4815
16	61.6	43.49644	43.86321	44.15403	131.5137	43.87775	44.04693	44.00009	131.9248	43.64117	44.12835	44.03235	131.8019	44.10606	44.25526	43.85365	132.215	43.94716	43.76655	43.9491	131.6628
17	61.7	43.47254	43.85442	44.24126	131.5682	43.92627	44.12329	43.85714	131.9067	43.75774	44.20879	44.15167	132.1182	44.1915	44.4143	43.86425	132.4701	43.94251	43.86392	43.92417	131.7306
18	61.8	43.42645	43.7618	44.23438	131.4226	43.91471	44.14454	43.75912	131.8184	43.74808	44.18239	44.22978	132.1603	44.06339	44.33672	43.80837	132.2085	43.90697	43.91742	43.89589	131.7203
19	61.9	43.39588	43.65442	44.12595	131.1763	43.83857	44.12253	43.82093	131.782	43.63668	44.04306	44.22927	131.909	43.83726	44.10941	43.73261	131.6793	43.8834	43.92624	43.83841	131.6481
20	62	43.40617	43.54357	43.96421	130.914	43.76083	44.06532	43.97397	131.8001	43.49578	43.85718	44.14601	131.499	43.70122	43.91832	43.62353	131.2431	43.87604	43.89347	43.7204	131.4899
21	62.1	43.4101	43.41872	43.81994	130.6488	43.74923	43.97167	44.02502	131.7459	43.3919	43.72023	43.99651	131.1086	43.6957	43.86741	43.4954	131.0585	43.85324	43.82445	43.59189	131.2696
22	62.2	43.35413	43.32913	43.72208	130.4053	43.77893	43.8756	43.87886	131.5334	43.35749	43.6704	43.83508	130.863	43.7198	43.8862	43.39986	131.0059	43.78505	43.74236	43.53447	131.0619
23	62.3	43.26973	43.36152	43.64659	130.2778	43.75318	43.81664	43.64223	131.212	43.39034	43.67681	43.73821	130.8054	43.7107	43.85523	43.35915	130.9251	43.67649	43.67607	43.55898	130.9115
24	62.4	43.23347	43.55564	43.57172	130.3608	43.65217	43.80202	43.51527	130.9695	43.44657	43.69049	43.74283	130.8799	43.70985	43.7726	43.34568	130.8281	43.59526	43.66018	43.59989	130.8553
25	62.5	43.27116	43.83588	43.51844	130.6255	43.56494	43.81542	43.5848	130.9652	43.4737	43.70137	43.78745	130.9625	43.75901	43.71842	43.35704	130.8345	43.61547	43.7118	43.60802	130.9353
26	62.6	43.34785	44.05586	43.52485	130.9286	43.55717	43.83464	43.73977	131.1316	43.45777	43.73837	43.77207	130.9682	43.80794	43.704	43.40843	130.9204	43.71366	43.7905	43.60519	131.1094
27	62.7	43.42161	44.0953	43.59604	131.1129	43.56592	43.84165	43.81166	131.2192	43.42252	43.83024	43.67624	130.929	43.79346	43.65791	43.47158	130.923	43.7828	43.81602	43.64971	131.2485
28	62.8	43.45574	43.94335	43.70224	131.1013	43.4971	43.8301	43.75021	131.0774	43.37717	43.95718	43.56604	130.9004	43.74665	43.54573	43.48417	130.7766	43.76999	43.75743	43.7536	131.281
29	62.9	43.4303	43.68957	43.79272	130.9126	43.37142	43.82389	43.63796	130.8333	43.30737	44.05496	43.50019	130.8625	43.74124	43.43347	43.42781	130.6025	43.71969	43.67403	43.86889	131.2626
30	63	43.37398	43.48449	43.82862	130.6871	43.30477	43.83976	43.5701	130.7146	43.22834	44.08305	43.47555	130.7869	43.78406	43.40866	43.36096	130.5537	43.68751	43.62709	43.92621	131.2408
31	63.1	43.34486	43.43234	43.81138	130.5886	43.36979	43.85725	43.58313	130.8102	43.20439	44.04075	43.48162	130.7268	43.8143	43.48249	43.34759	130.6444	43.69302	43.59056	43.86992	131.1535
32	63.2	43.3745	43.49195	43.7696	130.6361	43.51596	43.84276	43.67212	131.0308	43.27956	43.95089	43.52627	130.7567	43.79766	43.59101	43.37847	130.7672	43.72915	43.53406	43.69984	130.963
33	63.3	43.42456	43.50705	43.72219	130.6538	43.62604	43.78771	43.79499	131.2087	43.41122	43.8405	43.59971	130.8514	43.74664	43.64801	43.39775	130.7924	43.75142	43.50234	43.51546	130.7692
34	63.4	43.44059	43.39268	43.67466	130.5079	43.64026	43.71773	43.86401	131.222	43.49348	43.74751	43.65605	130.897	43.67313	43.62629	43.38828	130.6877	43.70207	43.55944	43.45161	130.7131
35	63.5	43.3886	43.24268	43.64442	130.2757	43.59836	43.66835	43.81815	131.0849	43.46881	43.69122	43.65195	130.812	43.59717	43.58216	43.4109	130.5902	43.59216	43.68077	43.54716	130.8201
36	63.6	43.29492	43.18452	43.66187	130.1413	43.56682	43.66167	43.70914	130.9376	43.39739	43.68644	43.60094	130.6848	43.54731	43.59478	43.50004	130.6421	43.51593	43.77335	43.70706	130.9963
37	63.7	43.22174	43.24739	43.71051	130.1796	43.56324	43.69759	43.632	130.8928	43.38415	43.74771	43.55937	130.6912	43.53989	43.66451	43.57217	130.7766	43.51787	43.77192	43.79526	131.085
38	63.8	43.21863	43.37333	43.72535	130.3173	43.55216	43.76859	43.60513	130.9259	43.42485	43.84332	43.5685	130.8367	43.55844	43.70393	43.50216	130.7645	43.53985	43.68407	43.75729	130.9812
39	63.9	43.28198	43.49854	43.66467	130.4452	43.52221	43.86162	43.578	130.9618	43.40821	43.88158	43.60151	130.8913	43.60477	43.65866	43.29652	130.5599	43.51832	43.57905	43.65266	130.75
40	64	43.34833	43.58066	43.56808	130.4971	43.51038	43.94946	43.53824	130.9981	43.27953	43.83401	43.59761	130.7112	43.68759	43.58206	43.10515	130.3748	43.48705	43.5294	43.59212	130.6086

Table S3

Data point	Mass (m/z)	w/ plasma_1			Integration	w/ plasma_2			Integration	w/ plasma_3			Integration	w/ plasma_4			Integration	w/ plasma_5			Integration
		1st	2nd	3rd		1st	2nd	3rd		1st	2nd	3rd		1st	2nd	3rd		1st	2nd	3rd	
0	60	44.04466	44.30421	44.07286	132.4217	44.05541	44.3929	44.28231	132.7306	44.10376	44.14164	44.48451	132.7299	44.01749	44.09772	44.69354	132.8088	44.52021	44.13453	44.03305	132.6878
1	60.1	43.79788	43.85856	43.96282	131.6193	43.77909	44.2689	43.90533	131.9533	43.73396	43.92971	44.23944	131.9031	43.92875	43.79448	44.51917	132.2424	44.13868	43.79393	43.89846	131.8311
2	60.2	43.70701	43.52716	43.84417	131.0783	43.52406	44.02251	43.65283	131.1994	43.48931	43.8124	44.06718	131.3689	43.81433	43.58035	44.22683	131.6215	43.82798	43.71936	43.80617	131.3535
3	60.3	43.74316	43.43382	43.76794	130.9449	43.40535	43.78191	43.53592	130.7232	43.47641	43.79206	43.91029	131.1788	43.74797	43.50643	43.98236	131.2368	43.65983	43.85855	43.74362	131.262
4	60.4	43.77267	43.55169	43.75576	131.0801	43.44564	43.68033	43.49475	130.6207	43.63907	43.87215	43.78642	131.2976	43.7688	43.56039	43.88945	131.2186	43.64411	44.02496	43.7384	131.4075
5	60.5	43.73811	43.77301	43.75774	131.2689	43.53335	43.68369	43.5091	130.7261	43.80764	44.03106	43.74162	131.5803	43.80394	43.67801	43.90853	131.3905	43.73443	44.11062	43.79597	131.641
6	60.6	43.70858	43.99374	43.7254	131.4277	43.54385	43.6852	43.5676	130.7967	43.84981	44.16574	43.71584	131.7314	43.76622	43.7435	43.95136	131.4611	43.86428	44.11153	43.86687	131.8427
7	60.7	43.75444	44.12476	43.66603	131.5452	43.47089	43.66155	43.58004	130.7125	43.75917	44.14761	43.57784	131.4846	43.67521	43.67795	43.98317	131.3363	43.96524	44.03374	43.87907	131.8781
8	60.8	43.83346	44.10047	43.61909	131.553	43.40596	43.65827	43.45118	130.5154	43.61805	43.92861	43.32854	130.8752	43.61817	43.54131	44.00665	131.1661	44.02779	43.86926	43.80412	131.7012
9	60.9	43.8546	43.92468	43.6158	131.3951	43.41625	43.67481	43.24393	130.335	43.52998	43.60244	43.18707	130.3195	43.64028	43.49418	44.01397	131.1484	44.11544	43.68608	43.6594	131.4609
10	61	43.81947	43.72698	43.67596	131.2224	43.50311	43.69675	43.17658	130.3764	43.57317	43.39027	43.38268	130.3461	43.72963	43.61064	44.02113	131.3614	44.28191	43.64243	43.53845	131.4628
11	61.1	43.84969	43.69748	43.80367	131.3508	43.66484	43.81185	43.45391	130.9306	43.77596	43.52195	43.92439	131.2223	43.8982	43.82611	44.12444	131.8487	44.50326	43.87603	43.63358	132.0129
12	61.2	44.06563	43.96466	43.99906	132.0294	43.95144	44.19303	44.10878	132.2532	44.12861	44.02548	44.64245	132.7965	44.24939	44.106	44.45481	132.8102	44.75682	44.39944	44.16941	133.3257
13	61.3	44.46718	44.51697	44.33096	133.3151	44.48809	44.94071	44.98618	134.415	44.64672	44.6845	45.42754	134.7588	44.87908	44.567	45.0398	134.4859	45.07402	45.11184	45.23048	135.4163
14	61.4	44.92921	45.21729	44.90977	135.0563	45.38855	45.96224	45.81881	137.1696	45.35909	45.32823	46.27079	136.9581	45.6898	45.28725	45.76388	136.7409	45.51709	45.88079	46.61725	138.0151
15	61.5	45.29735	45.85476	45.68212	136.8342	46.60207	47.00189	46.4037	140.0077	46.1635	46.04774	47.05349	139.2647	46.36865	46.10989	46.42688	138.9054	46.10796	46.57781	47.86213	140.5479
16	61.6	45.51357	46.24874	46.34253	138.1048	47.7901	47.76215	46.74025	142.2925	46.80132	46.92714	47.51582	141.2443	46.62946	46.76783	46.79881	140.1961	46.76505	47.11277	48.47589	142.3537
17	61.7	45.63308	46.34224	46.59994	138.5753	48.51458	48.0407	46.93039	143.4857	47.07045	47.67143	47.53286	142.2747	46.4256	47.15691	46.72344	140.3059	47.25231	47.44407	48.30335	142.9997
18	61.8	45.6916	46.1911	46.4213	138.304	48.53724	47.79585	46.9571	143.2902	46.98109	47.76664	47.23708	141.9848	45.94881	47.31852	46.26404	139.5314	47.27668	47.51969	47.59495	142.3913
19	61.9	45.60447	45.83919	45.94262	137.3863	47.88908	47.12233	46.68592	141.6973	46.61214	47.04124	46.76357	140.417	45.44078	47.21218	45.67295	138.3259	46.72297	47.22185	46.71276	140.6576
20	62	45.27441	45.27153	45.27816	135.8241	46.74794	46.18222	46.07421	139.0044	45.95485	45.87608	46.06137	137.8923	45.0207	46.69308	45.15793	136.8717	45.77927	46.4875	45.83854	138.1053
21	62.1	44.75734	44.5477	44.54969	133.8547	45.37693	45.16342	45.24719	135.7875	45.0569	44.81693	45.107	134.9808	44.65066	45.77034	44.73655	135.1576	44.80097	45.49237	45.0041	135.2974
22	62.2	44.24696	43.89955	43.94555	132.0921	44.14218	44.25229	44.3967	132.7912	44.17815	44.11684	44.15342	132.4484	44.26273	44.72681	44.35791	133.3475	44.06998	44.58578	44.27588	132.9316
23	62.3	43.88617	43.55518	43.61938	131.0607	43.37669	43.60318	43.69151	130.6714	43.62586	43.71263	43.56794	130.9064	43.89315	43.92548	44.03198	131.8506	43.67772	44.00541	43.77917	131.4623
24	62.4	43.66723	43.51685	43.5734	130.7575	43.15579	43.27658	43.26175	129.6941	43.48902	43.48676	43.45521	130.431	43.64151	43.51528	43.80886	130.9657	43.56658	43.74858	43.57644	130.8916
25	62.5	43.53144	43.58037	43.67688	130.7887	43.27315	43.22775	43.14759	129.6485	43.59075	43.41558	43.58422	130.5906	43.54356	43.39507	43.7088	130.6474	43.63892	43.70674	43.59434	130.94
26	62.6	43.47394	43.5859	43.76134	130.8212	43.44831	43.32653	43.28735	130.0622	43.70998	43.50264	43.67865	130.8913	43.53336	43.37961	43.72251	130.6355	43.80227	43.80973	43.66077	131.2728
27	62.7	43.52109	43.55264	43.71856	130.7923	43.52665	43.43584	43.52078	130.4833	43.74558	43.67975	43.65378	131.0791	43.52702	43.35976	43.79189	130.6787	43.9689	43.98777	43.65347	131.6101
28	62.8	43.63015	43.5672	43.57462	130.772	43.51654	43.46075	43.65771	130.635	43.71095	43.83035	43.55735	131.0987	43.49607	43.33444	43.8199	130.6504	44.06615	44.13503	43.58492	131.7861
29	62.9	43.7027	43.65581	43.45003	130.8085	43.48032	43.39365	43.59536	130.4693	43.63955	43.86845	43.43523	130.9432	43.46705	43.35363	43.74319	130.5639	44.07751	44.16016	43.54636	131.784
30	63	43.69571	43.77736	43.43628	130.9093	43.43445	43.30023	43.41232	130.147	43.55689	43.78482	43.34538	130.6871	43.46751	43.43285	43.59999	130.5004	44.02969	44.06149	43.58286	131.674
31	63.1	43.63554	43.87262	43.52306	131.0312	43.35436	43.2666	43.28936	129.9103	43.50319	43.63862	43.36488	130.5067	43.47685	43.51084	43.49614	130.4838	43.95467	43.91188	43.65187	131.5184
32	63.2	43.56336	43.89434	43.62628	131.084	43.25838	43.31242	43.31416	129.885	43.49645	43.5145	43.48317	130.4941	43.44937	43.50521	43.50294	130.4575	43.87574	43.80996	43.67297	131.3587
33	63.3	43.52731	43.81811	43.65205	130.9975	43.22996	43.38701	43.3998	130.0168	43.49745	43.43937	43.57371	130.5105	43.40113	43.42801	43.59599	130.4251	43.84407	43.79843	43.60877	131.2513
34	63.4	43.5751	43.6832	43.57854	130.8368	43.31171	43.4299	43.41538	130.157	43.46819	43.39025	43.53994	130.3984	43.4001	43.39093	43.69061	130.4816	43.8903	43.83558	43.51956	131.2454
35	63.5	43.69473	43.56947	43.47915	130.7433	43.43658	43.41835	43.34878	130.2037	43.42208	43.35587	43.42038	130.1983	43.46249	43.45868	43.7186	130.6398	43.96697	43.83612	43.5174	131.3205
36	63.6	43.78529	43.5292	43.44757	130.7621	43.49321	43.35436	43.30794	130.1555	43.39146	43.35971	43.31929	130.0705	43.50934	43.57881	43.68774	130.7759	44.00119	43.76789	43.6133	131.3824
37	63.7	43.75155	43.55611	43.48911	130.7968	43.43793	43.26619	43.37774	130.0819	43.38558	43.42567	43.30735	130.1186	43.47732	43.64126	43.65358	130.7722	43.99304	43.67561	43.68586	131.3545
38	63.8	43.61251	43.58717	43.51833	130.718	43.3143	43.23088	43.50662	130.0518	43.40287	43.54152	43.3917	130.3361	43.40065	43.59801	43.65255	130.6512	43.98082	43.62371	43.63411	131.2386
39	63.9	43.47102	43.56901	43.49304	130.5331	43.19903	43.29958	43.57979	130.0784	43.42601	43.65322	43.51601	130.5953	43.36635	43.48571	43.67793	130.53	43.96125	43.63101	43.50667	131.0989
40	64	43.39045	43.52202	43.48621	130.3987	43.15088	43.41828	43.55	130.1192	43.41538	43.69172	43.60199	130.7091	43.39193	43.38366	43.69733	130.4729	43.91018	43.67319	43.41922	131.0026

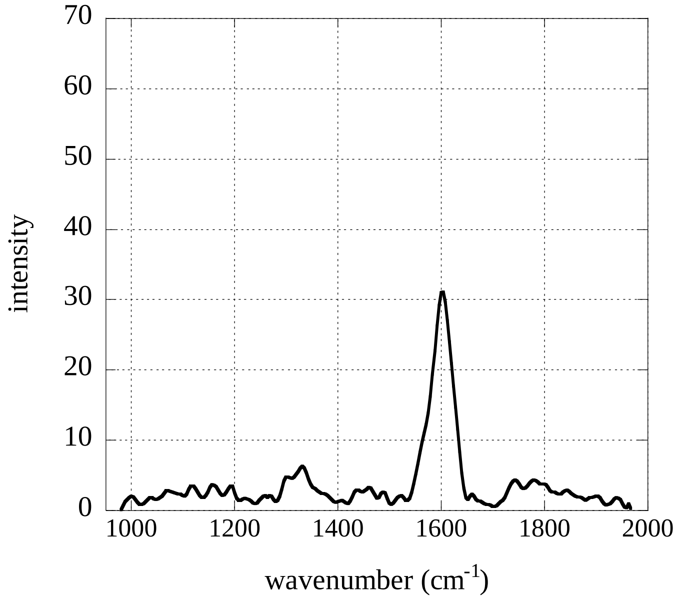

70
60
50
40
30
20
10
0
intensity
1000
1200
1400
1600
1800
2000
wavenumber (cm-1)

Figure S1

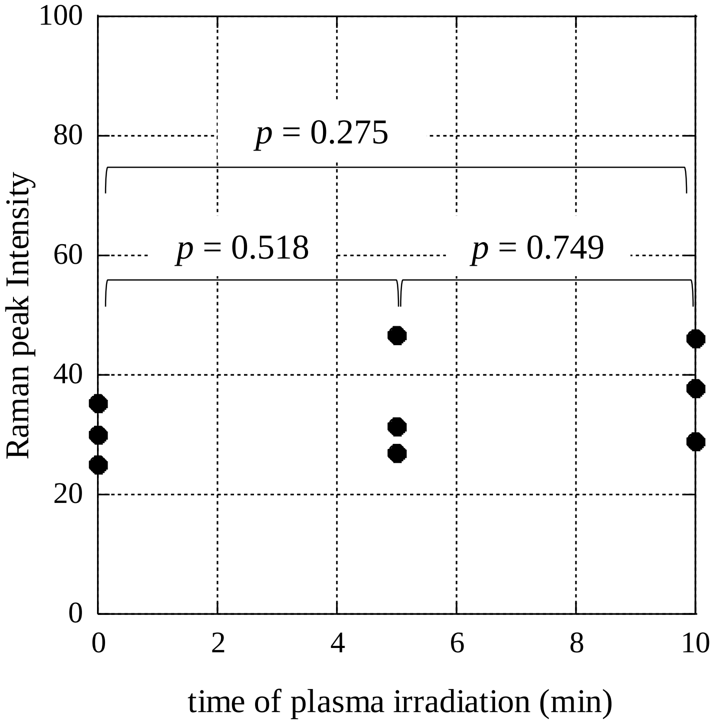

Figure S2

References

1. Piazza, G. J., Lora, J. H. & Garcia, R. A. Flocculation of high purity wheat straw soda lignin. *Bioresour. Technol.* **152**, 548–551 (2014).
